# The Role of Gut Microbiota in Intestinal Inflammation with Respect to Diet and Extrinsic Stressors

**DOI:** 10.3390/microorganisms7080271

**Published:** 2019-08-19

**Authors:** Stefani Lobionda, Panida Sittipo, Hyog Young Kwon, Yun Kyung Lee

**Affiliations:** Soonchunhyang Institute of Medi-Bio Science, Soonchunhyang University, Cheonan 31151, Korea

**Keywords:** gut microbiota, dysbiosis, diet, extrinsic stressors, intestinal inflammation, IBD

## Abstract

The gut microbiota maintains a symbiotic relationship with the host and regulates several important functions including host metabolism, immunity, and intestinal barrier function. Intestinal inflammation and inflammatory bowel disease (IBD) are commonly associated with dysbiosis of the gut microbiota. Alterations in the gut microbiota and associated changes in metabolites as well as disruptions in the intestinal barrier are evidence of the relationship between the gut microbiota and intestinal inflammation. Recent studies have found that many factors may alter the gut microbiota, with the effects of diet being commonly-studied. Extrinsic stressors, including environmental stressors, antibiotic exposure, sleep disturbance, physical activity, and psychological stress, may also play important roles in altering the composition of the gut microbiota. Herein, we discuss the roles of the gut microbiota in intestinal inflammation in relation to diet and other extrinsic stressors.

## 1. Introduction

Microorganisms reside on nearly all exposed surfaces of the human body and have co-evolved to develop mutualistic relationships with their hosts over millions of years [1]. The microbial community thrives within its host, maintaining a balanced composition and having roles in the maintenance of host homeostasis [2]. The gut microbiota, in particular, has shaped the development and function of the immune, metabolic, and nervous systems [3,4,5]. Thus, unnatural shifts in the composition of the gut microbiota, known as dysbiosis, can lead to several pathological disorders [6,7]. Intestinal diseases are especially prevalent in western societies. The increased incidence of these diseases suggests that specific factors including diet, environmental changes, antibiotic exposure, sleep disturbance, physical activity, and pathological stimuli can induce compositional shifts in the gut microbiota [8,9]. 

The primary role of the gut microbiota in the host centers on the digestion of indigestible nutrients. However, the microbiota is also involved in the absorption, metabolism, and storage of ingested nutrients, which may affect host physiology [10]. The composition of the gut microbiota gradually changes based on the host diet, eventually becoming a complex and established (or settled) microbial community [11]. Diet can determine the composition of gut microbiota, favoring the growth of organisms that are best suited for metabolizing commonly consumed food groups. Western diets are rich in a complex mixture of fats and are high in simple sugars, which significantly impacts the gut microbiome composition, and often leads to the development of gut inflammation and other related diseases, including intestinal disease [12]. Meanwhile, diets rich in prebiotics with high fiber content, such as those commonly observed in the vegetarian, Mediterranean, and in Fermentable Oligosaccharide Disaccharides Monosaccharides and Polyols (FODMAP) diets promote the growth of healthy intestinal microbiota, thereby reducing the risk of intestinal inflammation and diseases [10]. In addition, certain foods, such as fermented foods, can contain healthy microorganisms known as probiotics, which provide several benefits and can prevent and even cure intestinal inflammation and disease [13].

It has been increasingly recognized that factors other than diet can affect the composition of the gut microbiota and its function, particularly stress. Stress can be defined as a disruption in homeostasis due to extrinsic stressors, including environmental or physical stress, psychological stimuli, sleep disturbance or antibiotic exposure. Temperature changes, noise, hypobaria (decreasing barometric pressure on the body), or toxicant and pollutant exposure could be considered environmental stressors. In addition, trauma, stress from overcrowding, and psychological stress may affect the gut microbiota, leading to changes in the host physiology and further increasing disease risk. In contrast, physical activity, especially voluntary exercise, may provide several health benefits as it promotes the growth of healthy microbes and improves the host intestinal condition [8]. We live in a modern society, and as such, diets are often inconsistent and stress levels are higher. Both of these factors are correlated with increased risk of intestinal inflammation [8]. It has been suggested that these factors influence the composition and function of the gut microbiota and may lead to changes in its metabolites. These changes may then alter the immune response and compromise the host intestinal integrity, which can lead to the development of intestinal diseases. Therefore, it is important to understand the link between diet, extrinsic stressors, and the gut microbiota in order to prevent intestinal diseases.

## 2. The Gut Microbiota and Intestinal Inflammation

The phyla *Firmicutes* and *Bacteroidetes* are the most predominant phyla in a healthy gut, followed by *Actinobacteria*, *Proteobacteria*, and *Verrucomicrobia* [14,15]. Under normal conditions, the host immune system prevents the invasion of pathogenic bacteria while tolerating entry by normal gut microbiota [16]. If an imbalance in the gut microbiota occurs, the host immune system is altered, leading to the activation of an immune response which causes induction of a disease state [16,17]. In addition, dysbiosis increases the number of harmful bacteria in the gut [18], which may release enterotoxins, thereby increasing the permeability of the intestine, inducing the production of immunosuppressive proteins causing immune dysfunction, damaging intestinal epithelial cells, and affecting energy metabolism—leading to intestinal inflammation [17]. *Proteobacteria* species, belonging to the *Enterobacteriaceae* phylum, are commonly observed to be abundant in specific diseases, including inflammatory bowel disease (IBD) [15,19]. Intestinal inflammation provides an optimal environment for the growth of these organisms, consisting of aerobic conditions, biological sources from dying epithelial cells, and optimal mucus thickness. However, a bloom in *Enterobacteriaceae* species may not in itself induce disease states, but rather serve to increase susceptibility to intestinal inflammation and development of IBD [18]. 

IBD is a complex disease with both genetic and environmental causes [20]. Recent findings from a genetic perspective study identified 241 IBD susceptibility loci [21]. In addition, within a germ-free mouse model, the presence of enteric bacteria was reported as an important factor in the development of spontaneous colitis and activation of the immune system [22]. In general, enteric bacteria and normal gut microbiota can breach the intestinal barrier and induce intestinal inflammation in a susceptible host, suggesting that all bacteria have the potential to develop into pathogens [23]. Gut microbiota dysbiosis is also a key factor in the development of intestinal inflammation and IBD. Specifically, within IBD patients, a change in the ratio of *Firmicutes* and *Bacteroides* species has been reported [24]. *Ruminococcaceae* and *Lachnospiraceae* are the most common families of *Firmicutes* that were shown to decrease in IBD patients [25]. One of the most common bacterial species described as being closely associated with IBD is *Faecalibacterium prausnitzii,* affiliated with the family *Ruminococcacea,* which is also reduced under IBD conditions [26]. *F. prausnitzii* plays an important role in IBD through its secreted metabolite—butyrate [27]. They convert acetate, which is also produced by other species in the same family, such as *Ruminococcus albus*, which reduced under IBD conditions to butyrate [28,29]. Butyrate is essential in preventing the development of IBD as it facilitates the regeneration of colonocytes, thus maintaining intestinal integrity [30,31]. Moreover, butyrate also induces regulatory T cell (Treg) development to promote intestinal mucosal immune tolerance and maintain the balance between Th17 and Treg cells to prevent intestinal inflammation [27,32]. This evidence highlights the importance of the gut microbiota in intestinal disease. Whether the use of microbes in the treatment of IBD is effective, however, remains unclear. Clinical studies have been performed investigating the use of probiotics such as *Bifidobacterium* and *Lactobacillus* in the treatment of IBD patients [33]. Probiotics have subsequently been found to promote the secretion of anti-inflammatory factors which may aid the recovery of the intestinal barrier, improve the immune response, and protect against harmful bacteria resulting in the reduction of intestinal inflammation and mitigating the symptoms of IBD [17]. In summary, the gut microbiota plays an important role in the development of intestinal inflammation associated with IBD and is, thus, a key factor in the prevention and treatment of IBD. 

## 3. The Role of Diet and the Gut Microbiota in Intestinal Inflammation

Diet is one of many important factors which can affect the gut microbiota and many other aspects of human health and disease. The gut microbiota uses ingested nutrients as an energy source in fundamental biological processes, and changes in diet type or pattern may change the composition of the gut microbiota, as species more suited to metabolizing novel diet types will increase in population, while other species may become less abundance. These changes in the composition of the gut microbiota may affect host physiology and disease resistance [10]. 

### 3.1. Western Diets 

Western diets are associated with many diseases, including IBD, diabetes, obesity, hypercholesterolemia, and cardiovascular disease [12]. High fat-diets are used experimentally to mimic the effects of a western diet [34]. Fat is one of the most important macronutrients and is known to influence the composition of the gut microbiota, with *Bacteroides* often found at relatively low abundances, while *Firmicutes* and *Proteobacteria* are found at high abundances in patients consuming a high-fat diet [35]. High-fat diets are not only associated with metabolic inflammation but also with intestinal inflammation [36]. It is noted that the prolonged consumption of high-fat diets containing high amounts of saturated fat induces low-grade chronic intestinal inflammation in mice and increases IBD risk [37,38]. Alternatively, germ-free mice fed a high-fat diet did not show any signs of intestinal inflammation, demonstrating the interaction between high-fat diets and the intestinal microbiota in the development of intestinal inflammation [38]. High-fat diets alter the gut microbiota, leading to reduced tight junction protein expression, mucus production, antimicrobial peptide (lysozyme and RegIIIγ) production, and layer thickness [39]. Thus, the disruption of intestinal integrity and increase in bacterial translocation in the intestine induces intestinal inflammation [37,40]. 

**Figure 1:**
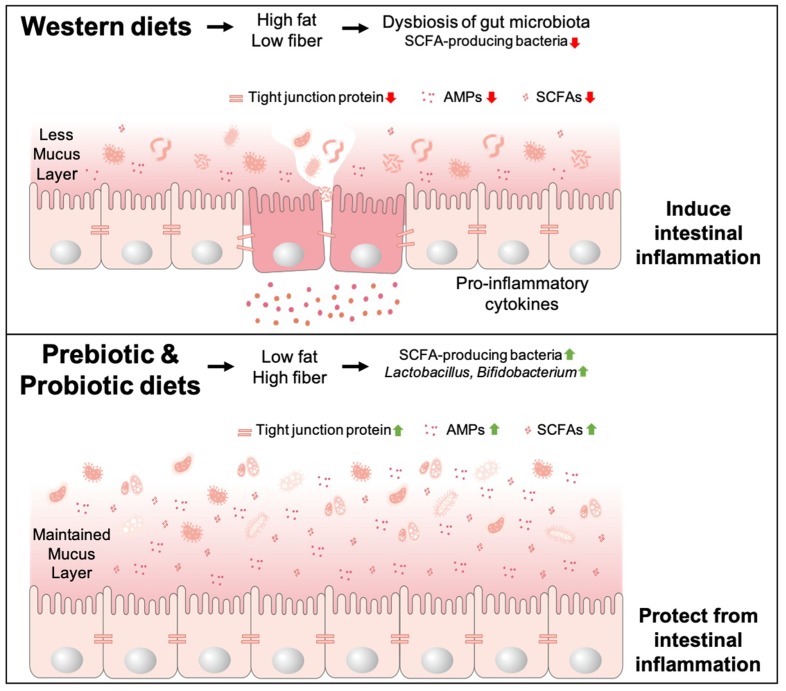
Diet alters gut microbiota associated with intestinal inflammation. Western diets containing high fat and lack of fiber changes gut microbiota population leading to a decrease of bacterial SCFAs, host AMPs, and mucus production as well as tight junction protein expression. Furthermore, it disrupts intestinal barrier, leads to bacterial translocation and increases pro-inflammatory cytokine production—resulting in intestinal inflammation. Meanwhile, prebiotic and probiotic diets provide a high fiber content, which increases the production of SCFAs, AMPs, mucus, and tight junction protein expression resulting in intact intestinal barrier and prevent from intestinal inflammation.

Inflammation in the intestine is caused by bacterial translocation, an association which has been confirmed by ex vivo experimentation, in which a culture of *Escherichia coli* stimulated the release of pro-inflammatory cytokines in mucosal samples from IBD patients [41]. However, specific microbial genera, such as *Lactobacillus*, *Bifidobacterium*, and *Faecalibacterium* may inhibit the inflammatory response in the intestine by reducing the expression of pro-inflammatory cytokines and stimulating the production of anti-inflammatory cytokines, which are generally decreased under high fat diet treatment [41,42,43]. Bacterial metabolites may mediate the beneficial role of these genera in the prevention of inflammation by altering microbial function. For example, the production of short chain fatty acids (SCFAs) was altered under a high fat diet as evident by decreased levels of butyrate [35,44]. Butyrate is one of the most important SCFAs in promoting the differentiation of colonic Tregs that play a role in maintaining intestinal homeostasis. In addition, butyrate also mediates the effects of Tregs through G-protein coupled receptors (GPRs), including GPR41, GPR43 and GPR109A, which are expressed in colonic and small intestinal Tregs [45]. However, the effects of a high-fat diet are not solely caused by its high fat content, but also by other dietary factors, such as a lack of fiber [34]. Free-fiber diets have been shown to exacerbate colitis [46]. In homeostasis conditions, bacterial dietary-fiber degradation and bacterial host-secreted mucus degradation are in a balance. However, free-fiber diets increase bacterial host-secreted mucus degradation, leading to disruption of the colonic mucus barrier and increasing host susceptibility to pathogenic infection (Figure 1) [46].

### 3.2. Prebiotic Diets

Vegetarian diets and Mediterranean diets typically have high dietary fiber contents, which constitute the primary sources of dietary microbiota-accessible carbohydrates (MACs) that can be digested by gut microbiota [10,47]. Among MACs, there is a specific subset of carbohydrates known as prebiotics that include indigestible oligosaccharides, and, more specifically, fructo-oligosaccharides. Prebiotics can enhance the growth of probiotics such as *Lactobacillus* and *Bifidobacterium* [48]. However, the decrease in the amount of available MACs induces a reduction in SCFA production, which benefits the host by serving as an energy source for inaccessible carbohydrate and potent regulatory molecules with physiological effects [10,49].

In animal models, fiber-supplemented diets consisting of short chain fructooligosacharides (SC-FOS) or short chain inulin-like fructans with degree 4 of polymerization (DP4), to a trinitrobenzene sulfonic acid (TNBS) model of rat colitis reduced intestinal inflammation, as was evidenced by reduced levels of TNF-α and NOS, while the proportion of *Lactobacillus* and *Bifidobacterium* increased [50,51,52]. In addition, administration of oral inulin reduced the severity of dextran sodium sulphate (DSS)-induced colitis in rats and increased proportions of *Lactobacillus* and *Bifidobacterium* in spontaneous colitis-developed transgenic rats treated with oligo-fructose enriched inulin (OF-IN) or FOS [53,54,55]. Moreover, supplementation of OF-IN to these transgenic rats also increased *Bifidobacterium* spp., decreased the level of pro-inflammatory cytokines and increased immunomodulatory molecules [55]. In summary, fiber-rich diets may protect from intestinal inflammation by increasing the production of SCFAs and the proportion of anti-inflammatory bacteria, including *Lactobacillus* and *Bifidobacterium*.

FODMAP diets are considered prebiotic diets, which serve as substrates for bacterial fermentation yielding SCFAs, including butyrate [56]. Moreover, diets that differ in their FODMAP content have shown to be associated with variation in fecal microbiota [56]. Ingestion of low FODMAP diets served to relieve irritable bowel syndrome (IBS) and decrease the total bacterial abundance including that of beneficial bacteria such as *Bifidobacterium*; butyrate-producing bacteria, such as *Lachnospiraceae*; and mucus degrading bacteria, including *Ruminococcus gnavus* and *Akkermansia muciniphila* [56,57,58]. Decreased carbohydrate consumption may explain the reduction in the abundance of these bacteria as they metabolize carbohydrates for their primary food sources [56]. It is, therefore, important to control the use of FODMAP diets in the asymptomatic population, while avoiding long-term consumption. IBS commonly presents in patients with IBD, while diets low in FODMAP have been shown to relieve IBS symptoms, the direct effect of FODMAPs in intestinal inflammation and IBD still needs to be addressed (Figure 1) [59,60].

### 3.3. Probiotic Diets

Several fermented foods contain probiotics (such as *Lactobacillus* and *Bifidobacterium*), including familiar food items such as kimchi, kefir, dry fermented sausage, yogurt, cheese, kombucha and miso, which contain viable cells in notable quantities ranging from 10^6^ to 10^9^ cells/g or cells/mL [13]. Probiotics are beneficial to host health by integrating into the gut microbiota and affecting its composition and activity through at least three different mechanisms [61]. They are able to stimulate the growth of resident bacteria by supplying metabolites such as SCFAs, vitamins, and other food sources produced via the degradation of mucin. Probiotics can directly impact the abundances of bacterial pathogens by decreasing pH, the production of lactate and SCFAs, niche competition, or through the production of exopolysaccharides (EPS) and bacteriocins. Some strains are also able to affect resident bacteria by interacting with the host epithelial immune system [61].

Lactic acid bacteria (LAB) are widely used in the production of fermented foods and are also found in the intestinal tract, as confirmed by the detection of *Lactobacillus* in human fecal samples [62]. *Lactobacillus gasseri* and *L. reuteri* proved to be true gut commensals while other species, such as *L. plantarum*, *L. rhamnosus*, and *L. paracasei* appear to be transient microbial taxa [63]. Probiotics may also play an anti-inflammatory role as clinical studies have found that probiotic consumption could be used to treat intestinal bowel disease [33]. The treatment of probiotic, including *L. reuteri*, *L. salvarius UCC118*, *B. infantis*, *L. plantarum 299v*, and *L. rhamnosus GG*, had beneficial effects in animal models [64]. Compromises in the integrity of the intestines may explain the development of intestinal diseases [40]. Therefore, the ability of probiotics to strengthen the intestinal barrier may provide some protection from those diseases. *Lactobacillus* has been reported to improve intestinal barrier function by modulating the expression of genes involved in tight junction signaling [65]. Treatment with VSL3 (a mixture of prebiotics and probiotics) promotes the expression of *MUC2* and mucus secretion which aid in strengthening the intestinal barrier [66].

The groups *Lactobacillus* (especially *L. reuteri*) and *Bifidobacterium animalis* subp. *lactic* produce proteins that promote mucus adhesion, referred to as mucus-binding proteins (MUBs), which enhance the interaction between probiotics and the host [67,68]. LABs can prevent pathogenic invasion by producing antimicrobial peptides (AMPs) such as bacteriocins, that can destroy pathogenic bacteria by forming pores in bacterial cell walls and inhibiting cell wall synthesis [69]. Moreover, probiotics modulate the microenvironment by producing lactic and acetic acids, which have antimicrobial effects and create an acidic environment, preventing the growth of bacterial pathogens [70,71]. The regulation of the host immune system and cytokine profile may be one of the major mechanisms by which probiotics are beneficial. The interactions between probiotics and the host immune system are visible in microbe-associated molecular patterns, including cell wall components such as polysaccharides, peptidoglycans, lipoproteins, and lipoteichoic acids that are recognized by pattern recognition receptors (PRRs) expressed in epithelial or host immune cells. One of the better-known PRRs that is triggered by *Lactobacillus* species is TLR2 (Figure 1) [72].

## 4. Extrinsic Stressors and The Gut Microbiota in Intestinal Inflammation

Extrinsic stressors are part of our daily life and can modulate the gut microbiota community composition and function. Extrinsic stressors can be classified into many categories depending on specific activities such as environment exposure, consumption of antibiotics, physical activity, sleep cycle, and psychological stimuli. These extrinsic stressors may interact with gut microbiota associated with intestinal inflammation.

### 4.1. Environmental Stressors

There are many bidirectional interactions between the gut microbiota and the host. Thus, the environmental factors that hosts are exposed to may affect the composition and function of the gut microbiota, leading to dysbiosis [73]. Several environmental factors can affect gut microbiota, including temperature, high altitude (HA), noise, toxicants, and pollutants [8].

Cold exposure in mice alters the composition of the gut microbiota. The transfer of bacteria from cold-exposed mice to germ-free mice partly affected intestinal structure, increasing the number of enteroendocrine cells (EECs) [74]. EECs are known to influence both the epithelial barrier and immune cells, while also having the ability to sense microbial metabolites and pathogen-associated molecular patterns (PAMPs) and secrete both peptide hormones and cytokines, which directly influence intestinal barrier function. In addition, mucosal immune cells have numerous receptors for peptide hormones which act as “cytokines” to modulate the activation of innate and adaptive immune cells [75]. Thus, EECs may play a significant role in intestinal inflammation. Moreover, cold exposure may alter the gut microbiota and is associated with intestinal inflammation through its effects on EECs. In fact, recent studies have reported associations between cold-exposed gut microbiota and EECs, however, whether the alteration of the gut microbiota directly regulates EEC function or increases the number of EECs remains to be determined. Furthermore, additional studies examining the mechanisms of how cold exposure affects the gut microbiota and its association with intestinal inflammation are required as well, as other mechanisms may better explain this association.

Heat exposure or heat stress in humans and rats has been found to increase pro-inflammatory cytokines, such as IL-1β, IL-6, and TNF-α which are known to decrease tight junction protein expression, including occludin, claudin-2, and ZO-1, respectively [76,77,78]. Furthermore, it may increase intestinal barrier dysfunction and can lead to the heightened permeability of the intestine to endotoxins, causing inflammation and sepsis [79]. The gut microbiota can also be affected by heat stress. The abundances of *Enterobacteria* (*Enterobacteriaceae)* and *Staphylococcus* were increased in the ileum of rats following heat exposure [80]. Moreover, an inflamed gut results in blooms of *Enterobacteriaceae* in patients with Crohn’s disease or ulcerative colitis, which can complicate the diagnosis of IBD [18]. Heat stress has two important factors that can cause intestinal inflammation and complicate IBD. Besides increases in intestinal barrier dysfunction by increase pro-inflammatory cytokines, heat stress also increases the abundances of *Enterobacteria*, leading to more complex intestinal inflammation and potentially to the development of IBD. However, further studies are still required to properly address the direct interaction among heat stress, the gut microbiota, and intestinal inflammation.

HA (around 3000 to 5000 m) exposure may lead to hypobaric hypoxia (HH). HH is a condition characterized by the reduced partial pressure of oxygen, which causes an oxygen imbalance in the tissues, potentially leading to severe physiological and psychological dysfunction in both humans and animals [81]. Few studies have reported the effects of HH on the composition of the gut microbiota; however, some studies have found that HH may cause intestinal dysfunction and trigger symptoms similar to those developed following HA exposure [82,83]. Furthermore, HH can damage the intestinal epithelial barrier by shortening the villi, widening tight junctions, reducing tight junction protein expression, and causing imbalances in the gut microbiota [84]. The decrease in beneficial bacteria, such as *Lactobacillus,* and the alteration in the gut microbiota may explain how HH causes intestinal barrier disruption and inflammation [84]. *Lactobacillus* is known to prevent intestinal inflammation by increasing the expression of tight junction protein, mucin secretion, and producing AMP to combat pathogenic invasion [65,66,69]. However, further studies are needed to elucidate the interaction between HA or HH and IBD with respect to the gut microbiota.

Environmental noise has many sources, including traffic, media, and household appliances. Long-term noise exposure is considered a health hazard and is also related to non-auditory-related diseases [85]. Chronic noise exposure in rats decreased the expression of intestinal tight junction proteins, increased pro-inflammatory cytokines in the intestine, and altered the relative abundances of gut microbiota, with a particular increase in the ratio of *Firmicutes* and *Bacteroides* [85,86]. Moreover, chronic noise exposure is also associated with a decrease in the abundance of *Faecalibacterium* spp. [85]. *F. prausnitzii*, one notable *Faecalibacterium* spp. and the most abundant butyrate-producing bacteria, has been associated with IBD, with marked decreases in the abundance of *F. prausnitzii* being observed in IBD patients [30]. *F. prausnitzii* provides energy to the colonocytes and maintains intestinal health by producing butyrate [30,31]. Moreover, the secreted metabolites of *F. prausnitzii* can block NF-κB and IL-8 in TNBS-induced colitis in experimental models [87]. These studies emphasize the importance of *F. prausnitzii* in protecting against intestinal inflammation through its effects on the intestinal barrier and in decreasing the expression of pro-inflammatory cytokines. Therefore, *F. prausnitzii* may be the fundamental link between chronic noise exposure and intestinal inflammation; however, further studies are needed to confirm this interaction and the possible involvement of another gut microbiota.

**Figure 2:**
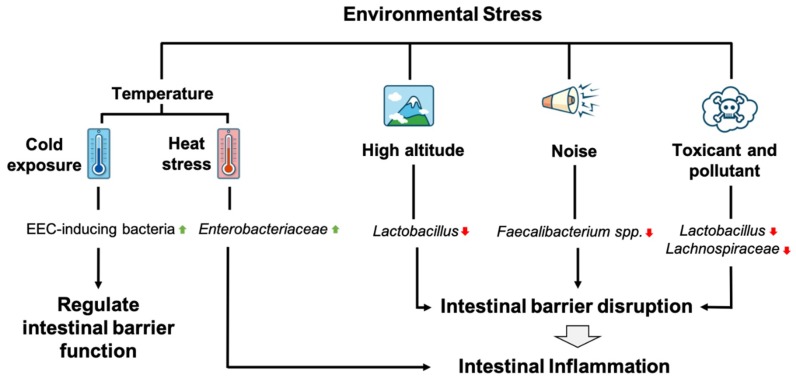
Alteration of gut microbiota by environmental stress impacts intestinal inflammation. Cold exposure changes the gut microbiota, which may be associated with the increase of EEC-inducing bacteria population and influence intestinal barrier function. Meanwhile, heat stress increases the population of *Enterobactericeae* and intestinal barrier disruption leading to intestinal inflammation. Other stressors such as high altitude, noise, pollutant and toxicant reduce anti-inflammatory gut microbiota— including *Lactobacillus*, *Faecalibacterium*, and *Lachnospiraceae*—increase the disruption of intestinal barrier, resulting in intestinal inflammation.

Exposure to environmental toxicants and pollutants is known to affect long-term systemic health and respiratory and cognitive ability [8]. The relationship between the gut microbiota and numerous diseases has been recently explored. The effects of toxicants and pollutants on disease may be mediated by the gut microbiota. Exposure to cadmium and lead decreases the abundance of *Lachnospiraceae* in the gut, which is often correlated with intestinal inflammation [11,88]. Chlorpyrifos is an organophosphate insecticide commonly used to treat fruit and vegetable crops and vineyards. Chronic exposure to low doses of chlorpyrifos increases the abundance of *Enterococcus* sp. and *Bacteroides* sp. while reducing the abundances of *Lactobacillus* sp. and *Bifidobacterium* sp. [11]. Polyaromatic hydrocarbons (PAHs) are persistent organic compounds that are highly resistant to environmental and biological degradation that can be bioaccumulated in organisms and are known as environmental and food contaminants [8,11]. Benzo[a]pyrene (B[a]p) is a PAH, oral ingestion of B[a]p increases intestinal inflammation and changes the composition of the gut microbiota in mice, decreasing the abundances of anti-inflammatory taxa including *Lactobacillus* and *Coprococcus* (*Lachnospiraceae*) and increasing the abundances of pro-inflammatory taxa, such as *Turicibacter* and *Clostridium*, which are associated with IBD [89]. *Lachnospiraceae* is a butyrate-producing bacterium that can convert lactate to butyrate, which is used by colonocytes in the maintenance of the intestinal barrier [31]. Moreover, butyrate can also induce the expression of Treg, which suppresses the colonic inflammatory response [32]. Therefore, decreases in the abundance of *Lachnospiraceae* may increase the risk of intestinal inflammation and IBD. Meanwhile, *Lactobacillus* is known to prevent intestinal inflammation by increasing the expression of tight junction protein, mucin secretion, and produces AMP, which combats pathogenic invasion [65,66,69]. In summary, environmental toxicants and pollutants change the composition of the gut microbiota, reducing the abundances of *Lachnospiraceae* and *Lactobacillus*, which may explain the effect of toxicants and pollutants to intestinal inflammation development. However, further study is needed to fully elucidate the mechanisms linking gut microbiota, toxicant and pollutant exposure, and intestinal inflammation (Figure 2).

### 4.2. Antibiotic Exposures

Antibiotics have been used to improve public health, agriculture, and medicine [90]. Studies have also examined the efficacy of treating IBD with antibiotics, especially for treating potentially harmful and invasive bacteria, such as *Enterobacteriaceae* [15]. The usage of antibiotic has been found to alter the taxonomic, genomic, and function of gut microbiota with potential persistent, long-term effects [90]. Antibiotic treatment disrupts microbial community composition, causing disturbances in species to species interactions. For example, mice treated with antibiotics have been shown to increase the host-derived free sialic acid in the gut which was further utilized by opportunistic pathogens such as *Salmonella typhimurium* and *Clostridium difficile* [91]. Moreover, antibiotics may have long-term effects on the composition of the gut microbiota; clarithromycin, metronidazole, and omeprazole were all found to have broad effects, yet the recovery was incomplete, and exhibited persistent effects for long periods of time [92]. Moreover, antibiotic treatment may induce resistance in gut bacteria, the genes for which can be transferred to other bacteria as is evidenced by an observed increase in *Enterococcus faecalis* and *Listeria monocytogenes* following the treatment of drinking water with tetracycline. *E. faecalis* was able to transfer the resistance genes to *L. monocytogenes* [93]. The alteration in the composition of gut microbiota and subsequent increase in the proportion of pathogenic bacteria, may further increase the risk of barrier disruption and severe intestinal inflammation [15]. In summary, antibiotic exposure may provide treatment for intestinal inflammation; however, it may also induce subsequent intestinal inflammation. Thus, further studies are required to better elucidate the effect of antibiotic exposure on gut microbiota associated with intestinal disease and inflammation [94].

### 4.3. Sleep Disturbance

Inadequate sleep has been associated with adverse physiological effects. In the rat model, sleep deprivation induced oxidative damage and cell death in several organs, including the liver, lungs, and small intestine [95]. Moreover, sleep deprivation resulted in pathogenic bacterial invasion to the mesenteric lymph nodes (MLNs) which is also associated with overgrowth of species such as *Enterobacter cloacae* and *Klebsiella pneumoniae* within the intestines. Immunosuppression and gut barrier dysfunction may facilitate the bacterial translocation from gut lumen to systemic circulation in sleep deprivation conditions [96]. Sleep deprivation has been associated with the incidence of IBD by increasing the level of pro-inflammatory cytokines, such as IL-1β, IL-6, TNF-α, and C-reactive protein which may result from bacterial translocation [97]. However, a study on sleep fragmentation in mice involving an increased food intake, resulted in an increased proportion *Lachnospiraceae* and *Ruminococcaceae* and a subsequent decrease in *Lactobacillaceae* spp., which is the opposite effect to what is observed in IBD in which *Lachnospiraceae* and *Ruminococcaceae* have been shown to be decreased [25,98]. Nevertheless, *Lachnospiraceae* has been associated with the attenuation of intestinal inflammation [31]. Therefore, further studies are needed to address the effect of sleep disturbance on gut microbiota composition in the development of intestinal inflammation.

### 4.4. Physical Activity

Exercise is considered an external factor that can regulate the gut microbiota. Exercise provides a range of health benefits, but is dependent upon the novelty, frequency, intensity, and duration of activity [8]. Voluntary running exercise in rats altered the composition of the gut microbiota; and increased levels of n-butyrate [99]. In addition, athletes also showed increase of SCFAs, potentially reducing the risk of intestinal inflammation [100]. Many recent studies using a range of animal models have found that exercise alters the composition of the microbiota regardless of the duration and type of exercise. Exercise increased the abundance of *F. prausnitzii*, which also provides protective effects against intestinal inflammation and IBD by producing butyrate [30,31,87,101]. Moreover, exercise increased the abundance of beneficial taxa, including *Lactobacillus* and *Bifidobacterium* [102,103,104], while reducing the abundance of potentially pathogenic taxa, including *Turicibacteraceae* and *Turicibacter* [105,106]. Germ-free mice were used to identify the role of the gut microbiota after exercise, confirming the beneficial effects of exercise in attenuating the effects of disease via the augmentation of colonic cytokine and transcription factor expression, which is known to be involved in regenerative tissue responses to colitis [107].

In contrast, intense exercise—which is common in athletes—negatively affects intestinal health through the disruption of the intestinal barrier by increasing body temperature. Intense exercise also directs blood away from the intestinal tract, which in combination with thermal damage harms the intestinal mucosa and the intestinal barrier. Moreover, prolonged exercise increases stress hormone levels and LPS translocation, resulting in increased pro-inflammatory cytokine levels and intestinal permeability [108,109]. The disproportionate increase of *R. gnavus,* one of the mucus degrading bacteria, has been associated with IBD incidence and may explain the disruption in the intestinal barrier allowing for increased bacterial translocation following intense exercise [106,110]. In addition, several gut microbiota species have been found to increase in athletes, including *Bacteroides*, *Veillonellaceae*, *Prevotella*, *Eubacterium*, *Ruminococcus*, and *Akkermansia muciniphila* [111,112,113]. Moreover, the abundance of *Prevotella* spp. has been positively correlated with IBD incidence. Alternatively, contradictory evidence has been presented regarding the effect of *A. muciniphila* on intestinal inflammation, with it being found to be reduced in IBD patients, and yet other studies reported it to exacerbate intestinal inflammation in the murine *Salmonella typhimurium* model and DSS-induced colitis model [110,114,115,116]. In summary, physical activity may provide advantages and disadvantages toward gut microbiota in intestinal inflammation. Therefore, further studies are needed to fully understand the interaction among physical activity, gut microbiota, and intestinal inflammation.

### 4.5. Psychological Stress

Psychological stress has been associated with multiple intestinal disorders and certainly affects the gut microbiota. Exposure to stressful stimuli in mice mimicking the symptoms of post-traumatic stress disorder (PTSD) in humans resulted in the alteration of the gut microbiota [117]. Other psychological stresses—including overcrowding stress—may affect the gut microbiota, increasing the abundances of *Staphylococcus*, *Streptococcus*, *Corynebacterium*, and *Enterobacteriaceae*. Furthermore, when psychological stress was more severe, the abundances of *Peptococcaceae*, *Bacteroidaceae*, and *Clostridium* also increased, while that of *Lactobacillus* was reduced [117,118,119,120]. Reduction in the abundance of *Lactobacillus* following exposure to stress can lead to intestinal inflammation. Alternatively, treatment with probiotics, such as *L. reuteri*, attenuated the development of intestinal inflammation following exposure to stress and immune challenge [121,122]. Indeed, exposing mice to a prolonged restraint stressor (stress stimuli) during an oral challenge with a colonic pathogen (*Citrobacter rodentium*) exacerbated colonic pathogen levels and intestinal inflammation [121,122]. This result was confirmed using germ-free mice colonized with microbiota from either stressor-exposed or non-stressed control mice. Mice colonized with the microbiota of stressor-exposed mice did not possess *Bifidobacterium*, whose heightened abundance can reduce the inflammatory response to *C. rodentium* [123].

Through an in silico study, it was found that alteration of the gut microbiota under stress reduced the biosynthesis and metabolism of fatty acids such as SCFAs. This may provide an additional explanation for the negative effects of stress following intestinal pathogenic challenge, as butyrate is well-known to prevent intestinal inflammation [124]. Moreover, stress exposure decreased the expression of mucin-2 and lysozyme, which may contribute to the dysbiosis of the gut microbiota by increasing the abundances of pro-inflammatory bacteria and reducing the abundances of butyrate-producing bacteria such as *Lachnospiraceae*. In addition, the transfer of gut microbiotas from stressor-exposed mice provided sufficient conditions to trigger the development of DSS-induced colitis [125]. In summary, psychological stress reduced anti-inflammatory bacteria; *Lactobacillus*, *Lachnospiraceae* and SFCAs which may increase the susceptibility toward intestinal inflammation and further IBD.

## 5. Conclusions

**Figure 3:**
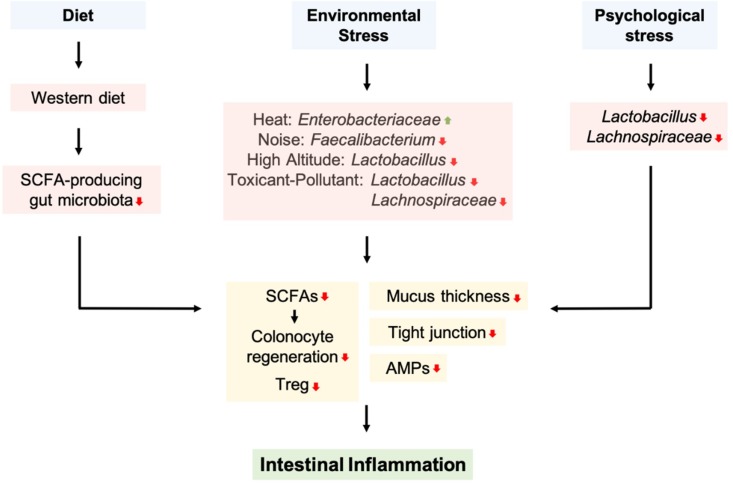
Diet and extrinsic stressors that alter the composition of gut microbiota and are associated with intestinal inflammation A western diet reduced SCFA-producing bacteria. Environmental stressors, including heat, noise, high altitude, toxicants, and pollutants decreased *Faecalibacterium*, *Lactobacillus*, and *Lachnospiraceae*, while increasing *Enterobacteriaceae*. In addition, psychological stress reduced the proportion of *Lactobacillus* and *Lachnospiraceae*. These gut microbiota alterations may decrease mucus, SCFAs, and AMPs production, as well as tight junction protein expression, resulting in intestinal inflammation.

Dysbiosis of the gut microbiota due to diet and extrinsic stressors, such as environmental factors, antibiotic exposure, sleep disturbance, physical activity, and psychological stresses, leads to alterations in intestinal microbiota composition and bacterial metabolite production, disruptions of host intestinal barrier integrity, and immune system, resulting in the development of intestinal inflammation and IBD (Figure 3). Therefore, it is important to understand the effects of diet and extrinsic stressors on the gut microbiota to prevent and cure intestinal inflammation. However, further research is required to fully address the direct interactions between diet, extrinsic stressors, and the gut microbiota in intestinal inflammation.
